# Stages of development and injury patterns in the early years: a population-based analysis

**DOI:** 10.1186/1471-2458-6-187

**Published:** 2006-07-18

**Authors:** Michael P Flavin, Suzanne M Dostaler, Kelly Simpson, Robert J Brison, William Pickett

**Affiliations:** 1 Department of Paediatrics, Queen's University, Doran 3, Kingston General Hospital, Kingston, Ontario, K7L 2V7, Canada; 2 Department of Community Health and Epidemiology, Queen's University, Abramsky Hall, Kingston, Ontario, K7L 3N6, Canada; 3 Department of Emergency Medicine, Queen's University, Empire 3, Kingston General Hospital, Kingston, Ontario, K7L 2V7, Canada

## Abstract

**Background:**

In Canada, there are many formal public health programs under development that aim to prevent injuries in the early years (e.g. 0–6). There are paradoxically no population-based studies that have examined patterns of injury by developmental stage among these young children. This represents a gap in the Canadian biomedical literature. The current population-based analysis explores external causes and consequences of injuries experienced by young children who present to the emergency department for assessment and treatment. This provides objective evidence about prevention priorities to be considered in anticipatory counseling and public health planning.

**Methods:**

Four complete years of data (1999–2002; n = 5876 cases) were reviewed from the Kingston sites of the Canadian Hospitals Injury Reporting and Prevention Program (CHIRPP), an ongoing injury surveillance initiative. Epidemiological analyses were used to characterize injury patterns within and across age groups (0–6 years) that corresponded to normative developmental stages.

**Results:**

The average annual rate of emergency department-attended childhood injury was 107 per 1000 (95% CI 91–123), with boys experiencing higher annual rates of injury than girls (122 vs. 91 per 1000; p < 0.05). External causes of injury changed substantially by developmental stage. This lead to the identification of four prevention priorities surrounding 1) the optimization of supervision; 2) limiting access to hazards; 3) protection from heights; and 4) anticipation of risks.

**Conclusion:**

This population-based injury surveillance analysis provides a strong evidence-base to inform and enhance anticipatory counseling and other public health efforts aimed at the prevention of childhood injury during the early years.

## Introduction

In Canada and other countries, injury is a leading cause of mortality and the most common cause of death among children [1-5]. There are approximately 10000 injury hospitalizations per year to Canadian children aged 1–9 years [6], and 200 injury-related fatalities representing one-third of all deaths in this age group [7]. Most pediatric injuries are of modest severity yet many can affect behavioural and emotional development [8]. Even minor injuries are stressful and treatment of these injuries imposes a financial burden on society.

Paediatricians, primary care physicians, nurses and public health professionals have important roles to play in the prevention of paediatric injuries. This is recognized by the existence of programs such as Safekids Worldwide [9] and the American Academy of Pediatrics' "The Injury Prevention Program" [10] which provides health care professionals and parents with age- and developmentally-appropriate resources for injury prevention [11]. In the early years, recognized developmental milestones and stages of growth [12] are associated with injury risks. In infancy, external causes of injury are related primarily to caregiver behaviour [13,14]. Through the preschool years, risks evolve as there is a shift from parent/caregiver-initiated activity to child-initiated activity. Although the caregiver maintains responsibility for a preschooler's environment, the child is capable of independently encountering situations that place them at risk.

Existing injury surveillance initiatives have examined the occurrence of paediatric injury in several contexts [1,2,11,13-22]. These include studies of hospital discharge and mortality records [1,20,21]; emergency department-based surveillance initiatives [2,14,15,19]; administrative data [22]; and injuries identified in primary care settings [11], or via caregiver surveys [13,16]. These studies have provided important information about the mechanisms associated with more serious injuries [1,20,21]; compared patterns of fatal versus non-fatal injuries [17,19]; and profiled specific external causes [15,18,19]. Most studies that have examined injury patterns by developmental stage have focused on injuries to infants [13,14,17,18]; or have employed broad (e.g. 5-year) age groups [1,2,11,16].

Our research setting in Kingston, Ontario is a Canadian Hospitals Injury Reporting and Prevention Program (CHIRPP) site [23]. The CHIRPP system in Kingston is unique because of its complete community coverage. We wished to understand the relationship between the child's developmental stage and the mechanism, nature and location of occurrence of injuries experienced by young children. No existing Canadian analyses address this topic and we could not find any population-based studies from other countries that examined injury patterns by developmental stage in an emergency department setting. This represents a major void in the biomedical literature. Our hope was that study findings would provide an evidence-base for prevention at the population level, and at the same time, provide useful guidance for anticipatory counselling.

## Methods

### CHIRPP

CHIRPP is an ongoing electronic injury surveillance program that operates in the emergency departments of 16 Canadian hospitals [23]. When an injured child is brought to the emergency department, the accompanying adult is asked to complete a self-administered questionnaire that details the mechanism of injury, its location of occurrence and the circumstances surrounding the injury event. The CHIRPP data collection process has been subject to formal validation efforts, and has been found to have a reasonable degree of accuracy for external causes of injury codes [24]. The clinical information is abstracted from the patient's medical chart including the nature of injury, body part(s) involved and treatment(s) received.

### Kingston CHIRPP

Kingston is a city in Eastern Ontario with a municipal population of 150000 [25]. It is home to two universities and a major health sciences complex. Kingston General Hospital (a regional trauma centre) and the Hotel Dieu Hospital have the only two emergency departments in the municipality. Hence, all injuries presenting to an emergency department are identified. The population-based nature of this surveillance system is unique as there are no other injury surveillance systems like this in Canada. Parents/guardians' descriptions of the injury circumstances are provided 80–85% of the time, remaining descriptions are abstracted from the medical chart. Repeat visits for the same injury event are excluded.

### Paediatric injury records

Records of all injuries occurring to children <7 years (0–83 months) were abstracted from the Kingston CHIRPP dataset. Records were limited to patients presenting from 1999–2002 (4 years) whose residential postal codes were within the catchment area. Available descriptors included: patient demographics (age in months, sex); nature and anatomical site(s) of injury; location of injury occurrence; and disposition from emergency. The CHIRPP categories of cause of injury ("breakdown event", "mechanism of injury", and "context") were used in combination with narrative descriptions of injury circumstances in order to classify external causes of injury.

### Age groups

Paediatric injury records were divided into age groups that were thought *a priori *to correspond with stages of growth and development in the early years: 0–11, 12–35, 36–59, and 60–83 months. Post-hoc analyses of three injury descriptors (external cause, nature, location) were conducted within narrower age groups to ensure that these four age groups were sufficient to capture salient injury patterns.

### Analysis

Overall and sex-specific rates of injury and associated 95% confidence intervals (CI) [26] were calculated per 1000 person-years by sex and then for each age group. Population projections [25] for 2000 (the study midpoint) were obtained for the hospital catchment area and used as denominators in these calculations[14]. Frequencies, cross-tabulations and χ^2 ^tests for linear trends in proportions were used to characterize injury patterns within and across developmental groups. Data management was conducted in Access 2000 (Microsoft, Richmond WA, 2000). Statistical analyses were performed in SPSS (v. 11.0, SPSS Inc, Chicago IL).

### Ethics

Institutional ethics approval for the injury surveillance program and this analysis were obtained from the Health Sciences Research Ethics Board at Queen's University.

## Results

### Paediatric injury rates

5876 injury records were included, an average of 1464 injuries/year or 107/1000/year (95% CI 91–123). Average annual age specific rates per 1000 children were as follows: 0–11 months 62 (95% CI 17–107); 12–35 months 153 (95% CI 122–184); 36–59 months 105 (95% CI 76–134); and 60–83 months 90 (95% CI 62–119). The estimated annual rate was higher for boys (122/1000; 95% CI 111–133) than for girls (91/1000; 95% CI 80–103). This sex-specific difference was noted in each of the four age categories, but was highest in the 12–35 month category (annual rate for boys: 174/1000; 95% CI 153–196; annual rate for girls: 133/1000; 95% CI 111–155).

### Age groups

**Figure 1:**
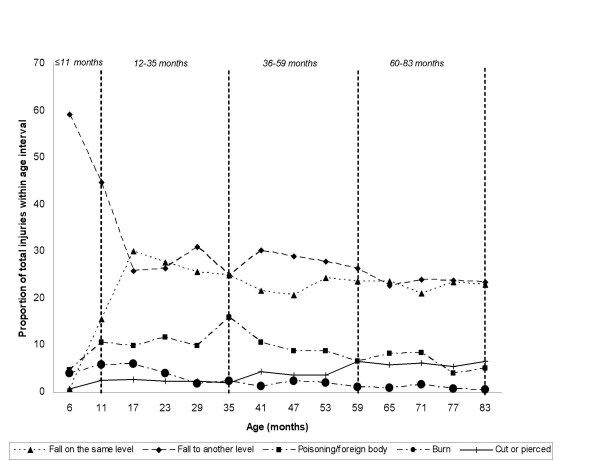
Profile of common external causes of paediatric injury within the early years.

The four age groups selected *a priori *to depict development stages and associated injury patterns were reasonable. While narrower groupings of age are possible with our data, observed injury patterns by external cause, nature, and location were consistent within these age groups. For example, Figure 1 presents the profile of the leading external causes by 6 month age intervals, and demonstrates the basic consistency of injury patterns within the selected age groupings.

### External causes of injury

Falls were responsible for 54% of all injuries (3141/5876) and of these, 3% (94/3141) were admitted (Table 1). Falls from or into furniture, on stairs, and from being dropped by another person were proportionally highest in the youngest age groups. Falls from playground equipment, during sports and from being pushed or shoved by another person were more common in the older age groups.

Poisoning, ingestion and foreign body injuries were collectively the second most frequent injury type overall (593/5876; 10%). Seventy-five children (52% of poisonings) ingested medications: the majority of these (63/75; 84%) were adult medications and 48/75 (64%) were by toddlers aged 12–35 months. The age-specific rate for poisoning by adult medication was 3.5 per 1000 per year for toddlers, a risk three times greater than for the next highest risk group, the 36 to 53 month olds. An additional 7% (143/2097) of the injury visits among toddlers were for "pulled arms", none of which required hospital admission.

A number of injury patterns (e.g. collisions, being struck by, pedal cycle collisions) increased in older age groups. Burns were highest among infants (0–11 months), and generally decreased with increasing age. There were 47 burns from contact with hot liquids in the two youngest age groups (0–11, 12–35 months), only three of these were bathroom scalds.

### Nature of injury

**Figure 2:**
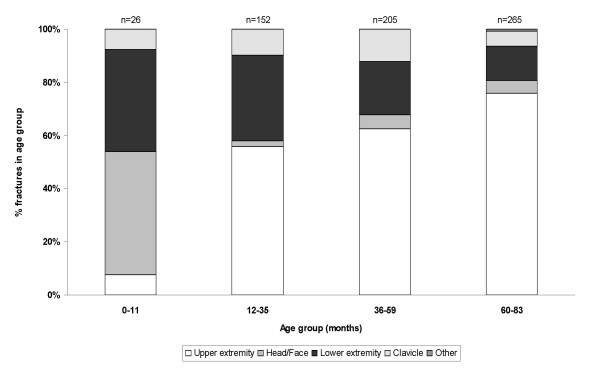
Anatomic location of fracture by age group

There were substantial variations by age group in the most frequent natures of injury (Table 2), although leading diagnoses were consistent by age group. By proportion, head injuries were most often observed among infants (98/424; 23%), while open wounds (589/1573; 37%) and fractures (265/1537; 17%) were more frequently represented in the 60–83 month age group. Head/facial and lower extremity fractures were common among infants, while upper extremity fractures were predominantly seen in the older age groups (Figure 2).

### Location of injury occurrence

The majority (3749/5876; 64%) of injuries occurred in home locations. Home injuries decreased with increasing age, while injuries in school/daycare environments, public parks, on the highway or road, and in sports/recreational facilities were proportionally more common in the older age groups.

### Disposition from emergency

Overall, 3064/5876 (52%) injuries were treated with follow-up as necessary; 1563/5876 (27%) were treated with follow-up required; and 166/5876 (3%) were admitted to hospital. 35.5% (154/434) of the youngest age group received "advice only" from the attending physician; this declined to 10.3% (163/1587) among the oldest age group. There were no fatalities recorded. However, in the catchment area, 5 deaths of children 0–6 years of age were determined to be trauma-related during the study period. Of these, 2 were unintentional (personal communication JL, Coroner's office, Ontario).

## Discussion

Young children are injured frequently. Our study is unique among emergency department studies of paediatric trauma because it is population-based and based upon a large (n = 5876) case series developed over four years. Patterns of injury we observed in our study are consistent with reports from others; for example, a recent profile of external causes of pediatric injuries leading to hospitalizations or death [21], and a large population-based analysis of the natures of medically treated injury developed from administrative data [22]. Our specific focus was to identify common patterns of injury by developmental stage and suggest evidence-based priorities for prevention. The audience was paediatricians, primary care physicians, nurses and public health professionals involved in the development of injury prevention efforts for the very young.

The observed injury patterns (Table 3) show that young children are exposed to varying hazards, with varying vulnerabilities which are influenced by physical and cognitive-social characteristics of different stages of development. Indicators of physical development include a variety of constructs such as height, reach, strength, and dexterity. Cognitive/social development can be depicted via an array of indicators such as curiosity, judgment, and ability to recognize hazards. With each new developmental stage, the profiles of physical and cognitive/social development change and new injury hazards emerge. Through awareness of how this occurs on a population basis, risks may be anticipated and avoided. Four priorities emerged from our analyses.

### Priority 1. Supervision

Young children are vulnerable to many injury hazards. They have limited ability to recognize danger and to foresee the consequences of their actions. Hence, adults have a responsibility for the protection of children by providing an appropriate level of supervision [27,28]. Morrongiello *et al*. [29] suggested that parental supervision is an essential element for the prevention of home injuries to toddlers. Optimal supervision was defined as that which keeps the caregiver "within both sight and reach" of the child. It has three essential dimensions: attention, proximity and continuity [30]. There is also a complex interaction between the child's physical health, social competence and risk-taking behaviour, and approaches to supervision should change with various combinations of these states [31]. Caregivers need to be aware of this and adjust supervision levels accordingly.

### Priority 2. Access to hazards

Conceptually, the most effective way of preventing an injury to a child is to keep him/her separated from the hazard in question. A child will not fall down stairs if there is a gate in place to prevent this access; they will not be burned if hot objects are inaccessible; a locked cabinet for drugs and noxious household chemicals will reduce ingestion risk. As infants' dexterity improves and they master grasping and releasing, anything within reach is a potential ingestion or choking hazard. Until the curious toddler learns what is unsafe to consume, access to visually attractive substances such as adult medications must be restricted. Animated play amongst toddlers and pre-schoolers in a physical space that has many hard edges and sharp corners is likely to result in injury. Caregivers need to adjust play areas accordingly and keep them free of known hazards.

While acknowledging that children learn to a great extent by exploring their physical environment, restricted access to chemical and physical hazards is needed. Passive safety barriers that are effective when a child is young [9,32] may lose their potential to prevent injuries as children age [33]. Caregivers must continuously re-assess the ability of "child proofing" barriers and restraints to serve their intended purpose.

### Priority 3. Falls from heights

Falls from one level to another are responsible for a large proportion of the observed injuries. Head injuries are common among the youngest children because they do not have the ability to shield themselves during a fall. Conversely, fractures of the upper extremity are leading diagnoses associated with falls from heights in older children due to the use of arms to protect the face. As children spend more time outside of the home, injury prevention shifts to child-centered strategies. As growing children appropriately gain more confidence in their physical abilities, the playground, ironically designed to enrich their development, becomes a hazard. Close and continual supervision remains important to ensure children follow safe rules for play. Secondary prevention measures are also important: adhering to playground equipment standards, and where there are high play structures, well-maintained soft or yielding ground surfaces become a priority [34-36].

### Priority 4. Anticipating risks

Children change dramatically during the early years. Advancing physical abilities often precede their ability to understand the consequences of their actions. We hypothesize that caregiver knowledge of growth and developmental milestones may reduce the likelihood that they will misjudge the ability of the child and adopt an inappropriate supervision strategy. With knowledge of injury patterns, the caregiver may be better equipped to help the child transition from a protected environment to acquiring an awareness of hazards.

In acknowledging that overprotection may stifle a child's development, a balanced approach to injury control is suggested. We expect that the caregiver can minimize exposure to hazards while providing appropriate supervision and ensuring that the child learns what is hazardous and complies with safety rules.

### Limitations and strengths

The injury rates presented in this study are limited to emergency department encounters. In the United States, a large proportion (7.5%) of office practice visits by children 19 and under are injury-related and as many as two thirds of all injury visits are seen in the primary care setting [11]. A large population based study using administrative data from Alberta, Canada found that 46% of medically treated injuries in children under 10 years of age were seen in physician's offices [22]. In our area, it is not possible to determine the number of children treated in physician's offices or outpatient clinics. Our rates underestimate the actual burden of paediatric injury, and may be overly inclusive of injuries requiring radiological assessment.

Descriptions of injury circumstances are based on self-reports collected as part of an established injury surveillance system. This system was not developed exclusively for the study of injury in children, but to monitor and describe patterns of injury in our entire community. This may limit detail in some situations and we are unable to identify intentional injuries from these descriptions.

Despite these recognized limitations, our study is one of the largest of its kind, it is population-based, and it provides a novel look at injury patterns in the context of an emergency department setting.

## Conclusion

Surveillance-based analyses are a necessary step in injury prevention as they identify recurring patterns and circumstances leading to injury. The findings of this study suggest the need for focused injury prevention initiatives tailored to particular stages of growth and development. The patterns and priorities identified here can be used to inform the adoption of evidence-based injury control efforts aimed at children of varying ages, including anticipatory counseling [37-39].

## Competing interests

The author(s) declare that they have no competing interests.

## Authors' contributions

MFP and WP were the key authors in the conception, design, drafting and coordination of the manuscript as well as the interpretation of the data. SMD participated in the design of the study, performed the analyses and helped to draft the manuscript. KS participated in the design and analysis of the data and the revision of the manuscript. RJB contributed substantively by revising the manuscript for intellectual content and participating in the interpretation of data and manuscript revision. All authors read and approved the final manuscript.

## Pre-publication history

The pre-publication history for this paper can be accessed here:



## Figures and Tables

**Table 1:** Population-based patterns among 5876 cases of paediatric injury: external causes across developmental groups

Age groups	0–11 months		12–35 months		36–59 months		60–83 months		All ages		Trend*	% admitted
Total number of emergency department-attended injuries	**434**		**2097**		**1758**		**1587**		**5876**			**2.8**
Annual injury rate per 1000 (95% CI)	62 (17–107)		153 (122–184)		105 (76–134)		90 (62–119)		107 (91–123)			

**EXTERNAL CAUSE OF INJURY**	n	%	n	%	n	%	n	%	n	%		

**Falls**	**262**	**60.4**	**1160**	**55.3**	**935**	**53.2**	**784**	**49.4**	**3141**	**53.5**	**↓**	**3.0**
												
***Falls from one level to another***	***212***	***48.8***	***569***	***27.1***	***501***	***28.5***	***374***	***23.6***	***1656***	***28.2***	**↓**	***4.3***
Fall from furniture	119	27.4	263	12.5	220	12.5	80	5.0	682	11.6	↓	3.2
Fall on stairs	35	8.1	169	8.1	93	5.3	51	3.2	348	5.9	↓	3.2
Fall from playground equipment (tree houses, trampolines, air castles)	2	0.5	45	2.1	104	5.9	162	10.2	313	5.3	↑	6.7
Fall from other specified object	20	4.6	68	3.2	73	4.2	70	4.4	231	3.9		4.3
Fall from being dropped	36	8.3	24	1.1	11	0.6	11	0.7	82	1.4	↓	9.8
												
***Falls on the same level***	***49***	***11.3***	***567***	***27.0***	***394***	***22.4***	***360***	***22.7***	***1370***	***23.3***		***1.3***
Fall into furniture	19	4.4	161	7.7	92	5.2	28	1.8	300	5.1	↓	
Fall into object	11	2.5	152	7.2	94	5.3	74	4.7	331	5.6		
Fall on same level-sports/pools	2	0.5	13	0.6	18	1.0	66	4.2	99	1.7	↑	
Other and unspecified falls	17	3.9	241	11.5	190	10.8	192	12.1	640	10.9	↑	
												
***Falls from collisions, pushing or shoving by/with another person***	***1***	***0.2***	***24***	***1.1***	***40***	***2.3***	***50***	***3.2***	***115***	***2.0***	↑	***3.5***
												
**Poisoning, ingestion, chemical spray, asphyxiation, foreign body**	**39**	**9.0**	**260**	**12.4**	**174**	**9.9**	**120**	**7.6**	**593**	**10.1**	**↓**	**4.7**
Mouth/ingestion, choking on food	33	7.6	166	7.9	63	3.6	26	1.6	288	4.9	**↓**	
Nose	0	0.0	45	2.1	47	2.7	15	0.9	107	1.8		
Eye/spray	1	0.2	24	1.1	27	1.5	28	1.8	80	1.4	↑	
Ear/ear lobe	1	0.2	9	0.4	18	1.0	29	1.8	57	1.0	↑	
Other foreign body	0	0.0	12	0.6	18	1.0	18	1.1	48	0.8	↑	
Inhalation smoke, carbon monoxide	4	0.9	4	0.2	1	0.1	4	0.3	13	0.2		
												
**Struck by object or person**	**30**	**6.9**	**90**	**4.3**	**106**	**6.0**	**154**	**9.7**	**380**	**6.5**	↑	**2.4**
Object fell, dropped on	15	3.5	52	2.5	32	1.8	32	2.0	131	2.2		
Object propelled, swung, moving	1	0.2	16	0.8	31	1.8	48	3.0	96	1.6	↑	
Person hit, kick, fall or step on	11	2.5	17	0.8	27	1.5	31	2.0	86	1.5		
Object/person sports	0	0.0	3	0.1	14	0.8	42	2.6	59	1.0	↑	
Other	3	0.7	2	0.1	2	0.1	1	0.1	8	0.1		
												
**Collisions with object/person**	**7**	**1.6**	**97**	**4.6**	**129**	**7.3**	**130**	**8.2**	**363**	**6.2**	↑	**1.4**
Collision with object	6	1.4	92	4.4	118	6.7	114	7.2	330	5.6	↑	
Collision with person	1	0.2	4	0.2	10	0.6	15	0.9	30	0.5	↑	
Collision other	0	0.0	1	0.0	1	0.1	1	0.1	3	0.1		
												
**Caught in or between**	**17**	**3.9**	**89**	**4.2**	**86**	**4.9**	**78**	**4.9**	**270**	**4.6**		**0.7**
												
**Cut or pierced**	**9**	**2.1**	**50**	**2.4**	**80**	**4.6**	**96**	**6.0**	**235**	**4.0**	↑	**0.9**
												
**Pulled arm**	**7**	**1.6**	**143**	**6.8**	**51**	**2.9**	**6**	**0.4**	**207**	**3.5**	**↓**	**0**
												
**Animal bite/scratch**	**7**	**1.6**	**41**	**2.0**	**57**	**3.2**	**59**	**3.7**	**164**	**2.8**	↑	**1.2**
												
**Pedal cycle injuries fell/lost control/collision (non motor vehicle)**	**0**	**0.0**	**13**	**0.6**	**42**	**2.4**	**82**	**5.2**	**137**	**2.3**	↑	**4.4**
												
**Burns**	**23**	**5.3**	**74**	**3.5**	**30**	**1.7**	**15**	**0.9**	**142**	**2.4**	**↓**	**4.9**
												
**Motor vehicle collisions**	**20**	**4.6**	**26**	**1.2**	**38**	**2.2**	**42**	**2.6**	**126**	**2.1**		**6.3**
Occupant	19	4.4	23	1.1	31	1.8	35	2.2	108	1.8		
Pedestrian/pedal cyclist	1	0.2	2	0.1	5	0.3	6	0.4	14	0.2		
ATV/snowmobile	0	0.0	1	0.0	2	0.1	1	0.1	4	0.1		
												
**Other/unspecified**	**13**	**3.0**	**54**	**2.6**	**30**	**1.7**	**21**	**1.3**	**118**	**2.0**		

* Results of significant Chi-square test for trend: ↑ proportion of total increased in older age groups (p < 0.05); **↓**proportion of total decreased in older age groups (p < 0.05)

**Table 2:** Population-based patterns among 5876 cases of paediatric injury: natures of injury by developmental groups

	0–11 months		12–35 months		36–59 months		60–83 months		Total		Trend*	%
Nature of Injury	N	%	N	%	N	%	N	%	N	%		admitted
Open wound	55	13.0	673	32.8	671	39.0	589	37.4	1988	34.5	↑	0.4
Superficial	84	19.8	269	13.1	233	13.5	229	14.6	815	14.1		0.2
Fractures	26	6.1	152	7.4	205	11.9	265	16.8	648	11.2	↑	14.7
Head injury	98	23.1	235	11.5	134	7.8	99	6.3	566	9.8	↓	2.8
Soft tissue injuries/sprain/strain	21	5.0	109	5.3	107	6.2	170	10.8	407	7.1	↑	1.0
Elbow pull/dislocation	14	3.3	186	9.1	84	4.9	5	0.3	289	5.0	↓	0
Foreign body ear, nose, respiratory, alimentary tract	13	3.1	81	3.9	78	4.5	40	2.5	212	3.7		3.3
Burns	24	5.7	71	3.5	31	1.8	15	1.0	141	2.4	↓	5.0
Bite	6	1.4	37	1.8	46	2.7	49	3.1	138	2.4	↑	1.4
Poisoning or toxic effect	12	2.8	92	4.5	32	1.9	8	0.5	144	2.5	↓	12.5
Eye injury/foreign body eye	1	0.2	30	1.5	34	2.0	48	3.1	113	2.0	↑	1.8
No injury detected	62	14.6	81	3.9	37	2.1	25	1.6	205	3.6	↓	0
Other	8	1.9	36	1.8	29	1.7	31	2.0	104	1.8		5.4

Total**	424	100	2052	100	1721	100	1573	100	5770	100		

* Results of significant Chi-square test for trend (p < 0.05): ↑ proportion of total increased in older age groups; **↓ **proportion of total decreased in older age groups

** excluding patients who left without being seen

**Table 3:** External causes of injury and key prevention opportunities inferred from population-based analysis

***Age Group***	**Key Injury Prevention Issues**		
	
Injury Mechanism	Nature of Injury	Developmental Aspect *(activity, behaviour, susceptibility)*	Prevention Priority*
***0–11 months***			
Falls	Head injury	Mobility (rolling), Vulnerability	1, 2, 3, 4
From furniture, being dropped	Open wound		
Foreign body ingestions	Ingestion	Grasping, Reaching, Curiosity	2, 4
Burns	Burn	Reaching, Curiosity, Vulnerability	2, 4

***12–35 months***			
Falls	Head injury	Mobility, Co-ordination, Balance, Curiosity,	1, 2, 3, 4
From/into furniture, on stairs	Open wound Superficial		
Foreign body ingestions	Poisoning	Mobility, Curiosity, Dexterity	2, 4

***36–59 months***			
Falls	Open wound	Mobility, Co-ordination, Curiosity	1, 2, 3, 4
From furniture, playground structures	Superficial Fracture		
Collisions with objects or persons	Fracture Open wound	Group Activity, Judgment	1, 4
Caught in/between objects	Open wound	Mobility, Judgment	2, 4

***60–83 months***			

Falls	Fracture	Mobility, Co-ordination, Curiosity	1, 2, 3, 4
From playground structures	Open wound		
Struck by objects or persons	Superficial Open wound	Group Activity, Judgment	1, 4
Cut or pierced by sharp object	Open wound	Dexterity, Judgment	2, 4

* 1. Supervision; 2. Access to hazards; 3. Falls from heights; 4. Anticipating risks

## References

[B1] Parkkari J, Kannus P, Niemi S, Koskinen S, Palvanen M, Vuori I, Jarvinen M (2000). Childhood deaths and injuries in Finland in 1971-1995. Int J Epidemiol.

[B2] Morrison A, Stone DH, Doraiswamy N, Ramsay L (1999). Injury surveillance in an accident and emergency department: a year in the life of CHIRPP. Arch Dis Child.

[B3] Bruce BS, Lake JP, Eden VA, Denney JC (2004). Children at risk of injury. Journal of Pediatric Nursing.

[B4] WHO (2001). Innocenti Report Card.

[B5] Public Health Agency of Canada Leading causes of death and hospitalization in Canada, 1997.

[B6] Canadian Instiute for Health Information National Trauma Registry 2004 Report Injury Hospitalizations (includes 2001/2002 data).

[B7] Statistics Canada (2005). Canadian Vital Statistics Death Database (2001/2002). http://www.statcan.ca/english/freepub/84-208-XIE/2002/tables.htm.

[B8] Rivara FP (1995). Developmental and behavioral issues in childhood injury prevention. J Dev Behav Pediatr.

[B9] (2005). Safekids Worldwide. http://www.safekids.org/.

[B10] Pediatrics AA (2005). The Injury Prevention Program. http://www.aap.org/family/tippmain.htm.

[B11] Hambidge SJ, Davidson AJ, Gonzales R, Steiner JF (2002). Epidemiology of Pediatric Injury-Related Primary Care Office Visits in the United States. Pediatrics.

[B12] Needlman RD, Behrman RE, Kliegman RM and Jenson HB (2004). Part II Growth and Development. Nelson Textbook of Pediatrics.

[B13] Warrington SA, Wright CM, Team ALSPACS (2001). Accidents and resulting injuries in premobile infants: data from the ALSPAC study. Arch Dis Child.

[B14] Pickett W, Streight S, Simpson K, Brison RJ (2003). Injuries Experienced by Infant Children: A Population-Based Epidemiological Analysis. Pediatrics.

[B15] Brownscombe J, Simpson N, Lenton S, Davis R, Barby T (2004). The potential of emergency department injury surveillance data: an illustration using descriptive analysis of data in 0-4 year olds from the Bath injury surveillance system. Child: Care, Health and Development.

[B16] Kohen DE, Soubhi H, Raina P (2000). Maternal reports of child injuries in Canada: trends and patterns by age and gender. Inj Prev.

[B17] Powell EC, Tanz RR (2002). Adjusting our view of injury risk: the burden of nonfatal injuries in infancy. Pediatrics.

[B18] Dedoukou X, Spyridopoulos T, Kedikoglou S, Alexe DM, Dessypris N, Petridou E (2004). Incidence and risk factors of fall injuries among infants:  a study in Greece. Arch Pediatr Adolesc Med.

[B19] Ballesteros MF, Schieber RA, Gilchrist J, Holmgreen P, Annest JL (2003). Differential ranking of causes of fatal versus non-fatal injuries among US children. Inj Prev.

[B20] Agran PF, Anderson C, Winn D, Trent R, Walton-Haynes L, Thayer S (2003). Rates of pediatric injuries by 3-month intervals for children 0 to 3 years of age. Pediatrics.

[B21] Agran PF, Winn D, Anderson C, Trent R, Walton-Haynes L (2001). Rates of pediatric and adolescent injuries by year of age. Pediatrics.

[B22] Spady DW, Saunders DL, Schopflocher DP, Svenson LW (2004). Patterns of injury in children: a population-based approach. Pediatrics.

[B23] Mackenzie SG, Pless IB (1999). CHIRPP: Canada's principal injury surveillance program. Inj Prev.

[B24] MacArthur C, Pless IB (1999). Evaluation of the quality of an injury surveillance system. Am J Epidemiol.

[B25] Statistics Canada (2001). Canadian Census of Population.

[B26] Rosner B (1990). Interval Estimation - Normal Theory Methods. Fundamentals of Biostatistics.

[B27] Dunne RG, Asher KN, Rivara FP (1992). Behavior and parental expectations of child pedestrians. Pediatrics.

[B28] Peterson L, Ewigman B, Kivlahan C (1993). Judgments regarding appropriate child supervision to prevent injury: the role of environmental risk and child age.. Child Development.

[B29] Morrongiello BA, Ondejko L, Littlejohn A (2004). Understanding toddlers' in-home injuries: I. Context, correlates, and determinants. J Pediatr Psychol.

[B30] Saluja G, Brenner R, Morrongiello BA, Haynie D, Rivera M, Cheng TL (2004). The role of supervision in child injury risk: definition, conceptual and measurement issues.. Injury Control and Safety Promotion.

[B31] Bradbury K, Janicke DM, Riley AW, Finney JW (1999). Predictors of unintentional injuries to school-age children seen in pediatric primary care. J Pediatr Psychol.

[B32] Harborview Injury Prevention and Research Center (2005). Best Practices:  Falls. http://depts.washington.edu/hiprc/practices/topic/falls/index.html.

[B33] Canada PHA (1997). Injuries associated with... BABY GATES. http://www.phac-aspc.gc.ca/injury-bles/chirpp/injrep-rapbles/gate3_e.html.

[B34] Mowat DL, Wang F, Pickett W, Brison RJ (1998). A case-control study of risk factors for playground injuries among children in Kingston and area. Inj Prev.

[B35] Laforest S, Robitaille Y, Lesage D, Dorval D (2001). Surface characteristics, equipment height, and the occurrence and severity of playground injuries. Inj Prev.

[B36] Howard AW, MacArthur C, Willan A, Rothman L, Moses-McKeag A, MacPherson AK (2005). The effect of safer play equipment on playground injury rates among school children. CMAJ.

[B37] Bass JL, Christoffel KK, Widome M, Boyle W, Scheidt P, Stanwick R, Roberts K (1993). Childhood injury prevention counseling in primary care settings: a critical review of the literature. Pediatrics.

[B38] Nelson CS, Higman SM, Sia C, McFarlane E, Fuddy L, Duggan AK (2005). Medical homes for at-risk children: parental reports of clinician-parent relationships, anticipatory guidance, and behavior changes. Pediatrics.

[B39] Committee on Injury and Poison Prevention (1994). Office-based counseling for injury prevention. Pediatrics.

